# Documentation of Complete Response in Metastatic Breast Cancer to Liver and Bone Achieved with Trastuzumab and Pegylated Liposomal Doxorubicin

**DOI:** 10.4137/cmo.s482

**Published:** 2008-06-11

**Authors:** Boris Kobrinsky, Eleni Andreopoulou, Karen Mourtzikos, Franco Muggia

**Affiliations:** New York University, New York, NY

## Never was Presented Elsewhere

We present a case report of a complete response to a combination of trastuzumab and pegylated liposomal doxorubicin in a female patient with recurrent metastatic HER-2 positive breast cancer after anthracycline-based adjuvant primary chemotherapy. To our knowledge this sequence of events has not previously been reported.

In September 2003, after routine mammographic detection, this 59-year-old business executive mother of two underwent right mastectomy and axillary node dissection with immediate reconstruction. Pathologic diagnosis included a T1N0M0 poorly differentiated infiltrating ductal carcinoma and further characterized by high proliferative fraction, negative hormone receptors, 3+ Her2 expression by immunohistochemistry, and 8-fold amplification of Her2 by FISH. Entry into Intergroup Study N9831 (1) was precluded by presumed pressure related brachial paresis during her postoperative recovery. Her adjuvant chemotherapy consisted of four cycles of doxorubicin (60 mg/m^2^) + cyclophosphamide (600 mg/m^2^) on a dose-dense (biweekly) schedule with G-CSF, followed by 7 doses of paclitaxel (100 mg/m^2^) every 10 to 11 days (without growth factors). She remained well for one and a half years, when lumbo-sacral and lower thoracic pain while exercising prompted a bone scan showing multiple lesions. PET/CT (Fig. 1a) confirmed bone scan findings and in addition showed liver involvement. Physical examination was unchanged, and cancer antigen (Ca 27.29) remained normal.

Treatment with trastuzumab (loading dose 8 mg/kg and subsequently 6 mg/kg) every three weeks, combined with pegylated liposomal doxorubicin (Doxil) 30 mg/m2 every three weeks was instituted, based on causing the least disruption of her daily activities, and on our institutional study (2,3). A baseline left ventricular ejection fraction by MUGA was 70% and unchanged 9 and 18 weeks later. Zoledronate 4 mg IV was added and repeated every 6–9 weeks. With appearance of grade 2 hand-foot skin changes after the third dose, Doxil was then given at a 6-week interval. A follow-up PET/CT shows bone sclerosis, with no uptake of 18F-fluoro-deoxyglucose (Fig. 1b). She will continued to receive trastuzumab 6 mg/kg every 3 weeks and Doxil 30 mg/m2 every 6 weeks in the absence of disease progression or adverse events.

Human epidermal growth factor receptor 2( HER2) gene amplification, found in 25% of primary breast tumors, is associated with an aggressive course and shortened overall survival (4) that has been known to improve with anthracycline-based therapy (5). Recently, this has been attributed to the frequent co-amplification of the topoisomerase 2 gene that is located in the same chromosome as Her2 (6). Trastuzumab, a humanized monoclonal antibody against the HER2 protein was shown in 2001 to significant improve the survival of women treated for HER2 overexpressing metastatic breast cancer when combined with doxorubicin and cyclophosphamide (first-line) or paclitaxel (second-line)(7). An unexpected finding was the enhancement of anthracycline (doxorubicin) cardiac toxicity by concomitant trastuzumab (8), and this combination was subsequently avoided, except in clinical studies evaluating combinations with the less cardiotoxic epirubicin (9) or the pegylated liposomal formulation Doxil (2,3). Therefore there was a clear rationale to investigate the safety and efficacy of this combination in the clinical setting. A 52% overall response rate and cardiac safety was reported with Doxil and Trastuzumab in 42 patients with overexpressing HER2 metastatic breast cancer (2).

Strategies for treatment of HER-2 positive breast cancer are evolving (10) and this is exemplified by this patient. In addition, this remarkable complete response (CR) by PET/CT suggests that such documented CRs may not only become more common, but offer a potential surrogate endpoint of comparative efficacy in the study of first-line or neoadjuvant regimens for this disease.

One year after onset of treatment, a routine repeat PET/CT documented reactivation of 18F-fluorodeoxyglucose (FDG) uptake in a 1 cm area in the left ischium. Assessment of the central nervous system by MRI documented 5 lesions in various areas of the cerebrum measuring from 2–3 mm without edema. Accordinly, in February 2007 she was treated with stereotactic (gamma-knife) radiation of these lesions, and she is being treated with capecitabine + lapatinib.

## Figures and Tables

**Figure 1a:**
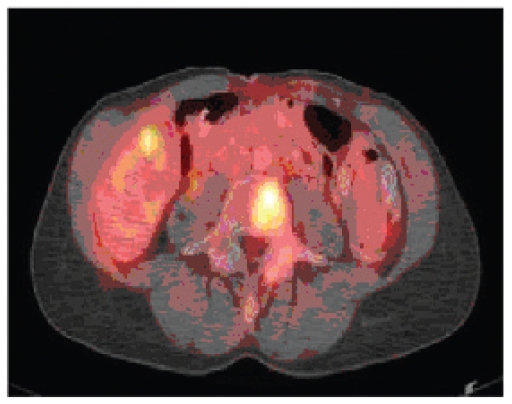
Before Treatment.

**Figure 1b:**
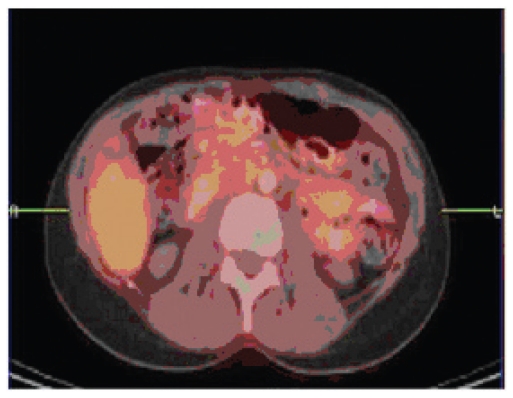
After Treatment.
